# Multiple species toxicity of selected platinum group of elements: focusing on *Hydra vulgaris* gene expression responses

**DOI:** 10.1007/s10646-025-02942-4

**Published:** 2025-07-29

**Authors:** J. Auclair, E. Roubeau-Dumont, P. Turcotte, C. Gagnon, F. Gagné

**Affiliations:** Environment and Climate Change Canada, Aquatic Contaminant Research Division, Montreal, QC Canada

**Keywords:** *Daphnia magna*, *Raphidocelis subcapitata*, *Hydra vulgaris*, Protein turnover/salvage, Oxidative stress, DNA repair

## Abstract

The aquatic toxicity of the most abundant platinum group of elements (PGE) was investigated using a multispecies test battery, and more specifically in *Hydra vulgaris* at the morphological and gene expression levels. Bacteria (*Aliivibrio fisheri*), algae (*Raphidocelis subcapitata)*, *Daphnia magna* and hydra were exposed to increasing concentrations of the following elements: iridium (Ir), palladium (Pd), platinum (Pt), rhodium (Rh) and ruthenium (Ru). The data revealed that the hydra and algae were more sensitive than the daphnids and the marine bacteria. In hydra, no lethal toxicity based on irreversible morphological changes was observed, however sublethal effects were noticed (tentacle budding, budding) with an EC20 at 10 µg/L for Ir, and Pd, Pt and Ru, EC20s at 20 µg/L. Rh produced no significant sublethal morphological changes. All tested PGE produced significant gene expression changes in pathways involved in protein turnover and degradation (ubiquitin and autophagy). Pd influenced genes at threshold concentrations reaching to <0.3 µg/L for protein turnover and degradation, oxidative stress, DNA repair and regeneration of 8-oxoguanosine, as well as for stem factor pathways. Rh, which was not (sub)lethally toxic based on morphology, influenced DNA repair of oxidized DNA and protein turnover pathways. In conclusion, PGE has the potential to alter protein turnover and induce oxidative DNA damage at environmentally relevant concentrations for receiving waters near wastewater discharges in urban area.

## Introduction

The platinum group of elements (PGE) consists of the following elements: osmium (Os), palladium (Pd), platinum (Pt), rhodium (Rh), iridium (Ir) and ruthenium (Ru). They are key elements for industrial processes and for therapeutics, mainly as antineoplastic agents (*i.e*. cisplatin, Pawlak et al., 2014). They are also used in catalytic converters, industrial alloys and dentistry (Lenz et al., 2005). PGE released by catalytic converters are considered as another source in the environment, containing Pd, Pt and Rh (Omrani et al., 2020). Emissions from automobiles occur mainly as particles <63 µm in the urban environment (Vaughan and Florence, 1992; Kumar et al., 2021). In the context of increased rainfall, an increase of deposited particles will leach into sewers and eventually be released into receiving waters and wastewater treatment plants, depending on their capacity to handle large inputs water. The automobile exhaust particles and the more soluble drugs find their way into the suspended matter fraction, sediments and water column (Rauch and Peucker-Ehrenbrink, 2015). It is estimated that only 10% of the total fraction of Pt is soluble, whereas for Pd and Rh, up to 50% of their total fraction is soluble (Pawlak et al., 2014). This suggests that a significant proportion of PGE could reach the aquatic biota. Moreover, in the context of the increasing population and aging, the occurrence of PGE from therapeutics (notably Pt) is likely to increase over time. On one hand, in river waters close to urban areas, PGE concentrations range from sub-ng/L reaching up to 200 ng/L for Pt and Pd (Abdulbur-Alfakhoury et al., 2021; Jackson et al., 2010). On the other hand, in hospital wastewaters supporting an oncologic ward in Vienna, levels of Pt ranged from 4.7 to 145 µg Pt/L, suggesting a regular input of these drugs in municipal wastewaters treatment plants, which are likely to increase from population aging as indicated above (Ghafuria et al., 2017). Pt-based drugs are more soluble and moderately removed (50–65%) by sludges (Lenz et al., 2005, 2007). Some elements were also found in predatory birds where Pd, Rh and Pt were consistently found in the blood, liver and eggs, suggesting an environmental exposure though the food chain (Ek et al., 2004). The lack of spatial trends suggests a widespread and diffuse distribution of PGE from non-point sources, such as automobile exhausts and long-range transport of micro/nanoparticles from road erosion runoffs (Ek et al., 2004). Ru is found in various alloys such as Pt/Ru electrodes of spark plugs, and is an important component of computer hard drives, which are usually disposed of in solid wastes. As such, these elements were detected in municipal effluents from major cities usually exposed to spark emissions and solid waste leachates, with concentrations reaching 200 ng/L for Pd and Pt (Jackson et al. 2010). The most abundant PGE were Pt, Pd, Rh and Ru compared to Ir and Os.

**Fig. 1:**
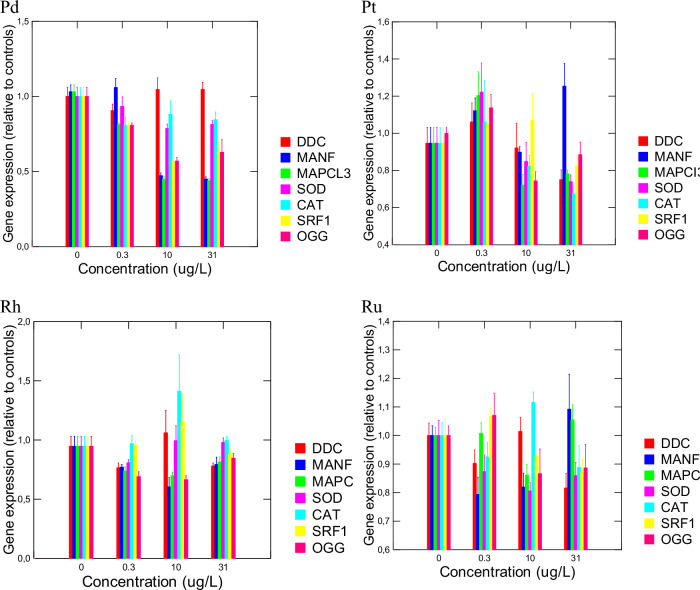
Gene expression changes in *Hydra vulgaris* exposed to platinum group of elements. The hydras were exposed to increasing concentration of Pd, Pt, Rh and Ru for 96 h at 20 °C. Gene expression was determined in hydra as described in the methods section. The data represent the mean with the standard error

The toxicity of PGE was examined using a “multitrophic” test battery encompassing bacteria (*Aliivibrio fisheri*), algae (*Raphidocelis subcapitata*) and daphnids (*Daphnia magna*) and *Hydra vulgaris*. This battery simulates a trophic chain where hydra feeds on daphnids, which feeds on algae. However, the luminescent bacterial test is of marine origin. Special attention was given to hydra, since it is a top predator and its reported sensitivity makes it a relevant invertebrate test species (Farley et al., 2025; Ghaskadbi, 2020) *Hydra vulgaris* Pallas, 1766 has been used for assessing the toxicity of environmental liquid chemicals and mixtures for more than 25 years (Fatima et al., 2024; Vimalkumar et al., 2022; Cera et al, 2020). This organism is considered a representative benthic species in freshwater environments and is ubiquitous in North America and Europe (https://inaturalist.ca/taxa/486293-Hydra-vulgaris). Moreover, recent studies revealed that this test species is highly sensitive to contaminants, making it a valuable screening test organism for environmental surveillance and compliance of effluents and leachates (Farley et al., 2025). The hydra belongs to the Hydrozoa class of Cnidaria phylum and is commonly found in freshwaters. They are solitary, sessile organisms found in temperate to tropical areas. The morphology of this organism is relatively simple, it is composed of a tubular body about 5–10 mm long with the base usually attached to a solid substrate (branches, stones and vegetation) and the head is composed of five to seven tentacles for feeding. They usually feed on small invertebrates such as water fleas and copepods. One attractive feature of hydra is that toxicity can be visually monitored by successive changes in morphology, as the severity (irreversible) of effects increases: tentacle retraction and budding, loss of tentacles, contraction of tubular body (tulip) and disintegration (Fig. 1S). The loss of tentacles, formation of tulip and disintegration stages are considered lethal and irreversible, while the first manifestations are reversible, hence considered sub-lethal (Fig. 1S). Recent evidence suggests that toxicity test with hydra could be used as alternative methods for fish toxicity testing (Dubé et al., 2019; Blaise et al., 2018). Indeed, Hydra was 5–10 times more sensitive to different rare earth elements and heavy metals than rainbow trout (Dubé et al., 2019). Because of the small size of hydra (1–10 mm), sublethal effects investigations at the molecular level need highly sensitive detection methods to compensate for the low amount of biomass. The development of a biomarker battery to better understand the long-term effects of contaminants to this organism are of value for risk assessment of chemicals and their mixtures. In respect to critical elements of technology such as PGE, the elucidation of the mode of action at the molecular and morpholigical levels are lacking in invertebrates at the present time. In the present study, the toxicity of Os was not investigated although this element is included in various catalytic alloys albeit at very low concentrations ( < 1 ng/L) and not detected in wastewaters (Jackson et al, 2010). However, the reported toxicity of Os in a freshwater ostracod was 11 µg/L (Khangarot and Das, 2009), concentrations yet found in wastewaters.

The purpose of this study was twofold: 1) examine the toxicity of selected PGE to hydra at both the morphological and gene expression levels, and 2) compare interspecific sensitivity with species from different trophic levels, *i.e*. bacteria, algae and invertebrates. Gene expression was analyzed using a very sensitive and quantitative reverse transcriptase polymerase gene expression methodology. The newly developed assay aimed at critical physiological targets, such as oxidative stress, neural activity, protein maintenance, DNA repair and cell regeneration and differentiation. Given the paucity of data in the literature, this information will provide new toxicological data for the PGE regarding the risk assessment community. Most studies examined the toxicity of PGE (namely Pt) in alga (Hourtané et al., 2024) and invertebrates such as nematodes, daphnids and mussels (Schertzinger et al., 2017; Zimmermann et al., 2017; Zimmermann and Sures, 2018).

## Methods

### Sample preparation

Analytical standard solutions of individual PGE -Ir(III), Ru(III), Rh(III), Pd (II) and Pt(IV)- were individually purchased from AnalytiChem (Canada). They were dissolved in 10% HCl at 10 mg/mL for most PGE, with the exception of Rh at 1 mg/mL. Exposure concentrations of the individual PGE were selected based on the reported solubility of critical elements of technology from toxicity studies with daphnids and zebra mussels (Zimmermann et al., 2017; Zimmermann and Sures, 2018; Vul’fson et al. 1990). At 100 µg/L in tap water, the recovery after 48 h of Pd, Pt and Rh were 87, 43 and 100%, respectively, suggesting that these elements remained mostly soluble albeit lower recovery for Pt. In the current study, 100 µg/L of each PGE in the hydra medium showed no apparent changes in turbidity nor precipitation after 1 h at the beginning of the exposures. Concentrations of each element were determined in the hydra medium after 1 and 96 h: samples were acidified with nitric acid (1%; Seastar grade) and analyzed by ion-coupled plasma mass spectrometry (ICP-MS, XSERIES 2 ICP-MS, Thermo Scientific, USA) with a detection limit of 0.005 µg/L. The instrument was calibrated with each element (1, 5, 25 and 50 ug/L). The following isotopes were used for quantitation to control mass interferences in standard mode: Ru101, Pb105, Ir193 et Pt195. For Rh103, the analysis was performed using kinetic energy discrimination mode.

### Aquatic toxicity bioassays

The toxicity of each of PGE (Ir, Pd, Pt, Rh and Ru) was evaluated using a multitrophic test battery: a luminescent marine bacterium commonly used for toxicity assessment (*Aliivibrio fischeri*), a freshwater algae growth/reproduction (*Raphidocelis subcapitata*), and the following 2 freshwater invertebrates: *Daphnia magna* and *Hydra vulgaris*. These assays were performed using standardized methodologies for bacteria (Environment Canada, 1992), algae (Environnement Canada, 2007), the water flea *Daphnia magna* (Environment Canada, 2000) and the hydranth *Hydra vulgaris* (Environment and Climate Change Canada, 2020). The bacteria test uses the bioluminescence properties of *Aliivibrio fisheri*, to assess survival, based on the decrease of bioluminescence measured after 15 min (the effective concentration that decrease luminescence by 50% (Effective Concentration 50% or EC50). The algal growth/reproduction bioassay determines the effective concentration that reduces algal growth by 50% (EC50) after 96 h of exposure. The *Daphnia magna* acute lethality test is based on the survival following 48 h of exposure to assess the lethal concentration at which 50% of the population is dead (LC50). Daphnid immobilization (absence of swimming activity) was also determined (EC50%). For all the bioassays, the maximum exposure concentrations was 100 µg/L based on the limited solubility of the PGE. The exposure experiments were repeated 3 times to ensure reproducibility.

The hydra bioassay determines the LC50 based on severe and irreversible morphological changes, such as loss of antenna/tentacles and severe contraction of the tube-like body, called the tulip stage, (see Fig. 1S, Blaise and Kusui, 1997). The sublethal effect (EC50) was based on observed reversible morphological changes: shortening of the tentacles and budding at the end. Hydra were reared in 1 mM CaCl_2_ containing 0.1 mM EDTA and 0.5 mM TES buffer pH 7.4 (Blaise et al., 2018). They were fed with fresh suspensions of *Artemia salina* each day, giving a doubling time of 4–5 days. At the beginning of exposure to PGE, adult hydra (3 individuals per 4 mL well in triplicate) were exposed to increasing concentrations of each of the PGE (0.3, 1, 10, 32 and 100 µg/L) in 4 mL total volume in 12 wells polystyrene microplates for 96 h. To ensure sufficient biomass for genomics, the assays were repeated in 2 additional microplates, each containing 3 wells per treatment containing 3 hydra per wells (total of 9 hydra per treatment). Hydras were not fed during the exposures. Given that the exposure time encompasses the reproduction period (appearance and release of polyps) after 4 days, this assay could be considered chronic for this species (Farley et al., 2025). At the end of the exposure experiments, the lethal and sublethal responses were determined using a 4–6 X stereomicroscope. The surviving hydra (without apparent morphological changes that were are deemed lethal) were collected and immediately stored in RNA stabilizing media (RNA later) at 4 °C for 24 h then stored at −20 °C for subsequent gene expression analyses as described below. The controls consisted of the hydra medium only. Given that Ir levels are very low in the environment ( < 7 pg/L range; Musil et al., 2023), the sublethal effects at the gene expression level were not determined.

### Gene expression analysis

Total RNA was extracted from N = 9 hydra using the RNeasy Plus Mini Kit (Qiagen) according to the manufacturer’s instructions. RNA concentration and purity were assessed with the NanoDrop 1000 (Thermo Fisher Scientific, ON, Canada), and RNA integrity was verified using the TapeStation 4150 system (Agilent) with the Agilent RNA ScreenTape Assay (cat # 5067-5576, Agilent Technologies Inc., Santa Clara, USA). Reverse transcription was performed with the QuantiTect® Reverse Transcription Kit (Qiagen), ensuring the complete removal of genomic DNA, following the manufacturer’s instructions. The resulting cDNA samples were stored at −80 °C until quantitative real-time PCR (qPCR) analysis.

All qPCR analyses were conducted using SsoFast™ EvaGreen® Supermix (Bio-Rad, Mississauga, ON, Canada) and the CFX96 Touch Real-Time PCR Detection System (Bio-Rad, Mississauga, ON, Canada). For each selected primer pair and gene identification (Table 1), a calibration curve (starting cDNA concentration: 10 ng, 8 serial dilutions) was generated, with PCR efficiency values ranging between 95 and 115%, and the limit of quantification was determined. Each reaction was run in duplicate and consisted of 5 µL cDNA, 6.5 µL of 2× SsoFast EvaGreen Supermix (Bio-Rad), 300 nM of each primer, and DEPC-treated water (Ambion) up to a total volume of 13 µL. Cycling parameters were as follows: 95 °C for 30 s, then 40 cycles of 95 °C for 5 s and 60 °C for 10 s for HPRT, RPLPO, Efα, DDC1, SRF, and OGG; 95 °C for 30 s, then 40 cycles of 95 °C for 5 s and 56 °C for 10 s for CAT and MANF; and 95 °C for 30 s, then 40 cycles of 95 °C for 5 s and 56 °C for 30 s for MAPLC3. Amplification specificity was verified with a melting curve. A no-template control (NTC) was included on each plate. Data analysis was performed using CFX Maestro (Bio-Rad). Gene expression data was normalized by 3 reference genes indicated in Table 1 (Livak and Schmittgen, 2001).

**Table 1:** Gene identity and primer sequence for expression determination

Function	Gene name	Forward/Reverse primers (5′— > 3′)	Amplicon (base pairs)
Housekeeping genes	hypoxanthine-guanine phosphoribosyltransferase-like *Hprt2*	GAA TTG AAC GCA TGG CTC GT/GTC TTG GCT GAA CCG AAA ACC	98
	60S acidic ribosomal protein P0-like *Rplp0-1*	CTG AGG CTG CTC TTC TTG CT/GGA CTG AAA ATG CTT CCG TTG T	94
	elongation factor-1 alpha *Ef1a-2*	TGC TCC TGG ACA TCG TGA CT/CAA CGA TGA GTA CGG CAC AAT C	77
Autophagy and Ub-pathway	Microtubule-associated protein 1 light chain 3 *Map1lc31*	CCA GAG AAA GCG AGA ATC CGA/TGG AGA GCA TAC CAA CTG TCA T	152
Stress and antiox.	Mesencephalic astrocyte-derived neurotrophic factor homolog *Manf-2*	CCA CTC GCA TAC TAC AAG CCT/ACA ACC ACT ACA AGT CTC ACC C	180
	superoxide dismutase [Cu-Zn]-like *Sod1-2*	ACC TGG TAA GCA CGG TTT TCA/ TGC ACC ACT CCA TCT TTA CCA	171
	catalase-like *Cat-1*	ACA GCC TCA ATG ACT GTT GGG/CCA CTC CAT TCA GAG CAG CC	196
DNA damage and repair	8-Oxoguanine DNA Glycosylase *Ogg1-1*	TGT GAC TGG AGT TGA AGA TGC T/ACT CCA GGC AAT GAG CAA AGA	174
Regeneration and Stem factor	Serum Response Factor *SRF1*	CTT GTG GCA TCG GAA ACA GG/TGC TTT GCC ACT TTC AGA GGT A	84
Neural activity	Dopa Decarboxylase *Ddc-1*	GCC CCA GTT GAG CCA GAT AA/ CAG TGA GTG ACA CCT GGC AT	77

### Data analysis

The LC50 and EC50/20 obtained from each of the bioassay were determined using the Spearman-Karber method (Finney, 1964). Toxicogenomic data were expressed as effect thresholds as defined: threshold = (highest no effect concentration x lowest significant effect concentration)^1/2^. The gene expression data were analyzed using a rank-based analysis of variance followed by the Conover-Iman test for differences from the controls. Possible relationships between toxicity and genes expression data were determined using the Spearman rank procedure. The biomarker data were also analyzed by hierarchical tree to determine similarity of effects between the elements using the Pearson correlation coefficient (1-r) as the metric distance between the observed effects. Significance was set at p < 0.05. All the statistical analyses were conducted using SYSTAT (version 13, USA).

## Results

The physico-chemical characteristics of the selected PGE were reported in Table S1. Pd, Rh and Ru have similar molecular weight (106.4–101.9 g/mol) while Ir and Pt are heavier (192.22 and 198.1 g/mol, respectively). The ionic radius varied between 64–84 pm, with Rh having the largest value. However, the electronegativity potential was similar among these elements, varying between 2.2–2.8 (Pauli’s scale). The redox potential was increased as follows Ru<Rh<Pd<Pt<Ir, suggesting the Pd, Pt and Ir are stronger oxidizing elements than Rh and Ru. The stability of these elements in the incubation media was examined without the presence of hydra after 1 and 96 h (Table S2) The data revealed that these elements were generally highly stable in solution over the exposure period, and the measured concentrations where close ( > 90%) to the nominal concentrations, except for Pd, where at 10 µg/L the measured concentration was 60% of the nominal concentration.

The toxicity of the selected PGE was examined using a multitrophic test battery (Table 2). No toxicity was observed in the marine bacteria *Aliivibrio fisheri* for all tested elements. They were however toxic to algae, at concentrations ranging from 19 µg/L to 67 µg/L in the following order: Pd~Rh<Ru<Ir. Pd was the only toxic element for *Daphnia magna*, with an LC50 and EC50 (swimming inhibition) averaged at 26 µg/L after 2 days. Finally, although no lethal effects were observed in Hydra (LC50 > 100 µg/L), all tested elements but Rh produced sublethal effects based on morphology. The sublethal toxicity of the selected PGE in hydra was similar, with an average EC20 of 20 µg/L. The preponderance of sublethal morphological effects in hydra with most of the tested elements prompted examination of the sublethal effects based on gene expression with newly developed gene targets in Hydra (Table 1).

**Table 2:** Toxicity data of platinum group of elements based viability (LC50) and morphology (EC50 and EC20)

Elements	*Aliivibrio fisheri* (Microtox) *LC50 LC25* (µg/L)	*Hydra vulgaris*^*a*^ *LC50 EC50 EC20* (µg/L)			*Daphnia magna*^*b*^ *LC50 EC50* (µg/L)		*Raphidocelis subcapitata* *CI25 CI50* (µg/L)	
Platinum	>100 > 100	>100 (28–115)^c^	50 (6–36)	21	>100	>100	>100	21 (12–64)
Palladium	>100 > 100	>100 (31–60)^a^	44 (19–27)	23	26 (15–42)	26 (15–42)	6.2 (5–7)	13 (11–15)
Rhodium	>100 > 100	>100	>100	>100	>100	>100	7 (5.7–9)	19 (15–24)
Iridium	>100 > 100	>100	>100	10 (2–71)	>100	>100	86 (76–96)	>100
Ruthenium	>100 > 100	>100	>100	19 (9–39)	>100	>100	37 (33–40)	67 (53–75)

^a^*Hydra vulgaris*: LC50 based on irreversible damage (tulip stage-mortality); EC50 or EC20: based on reversible damage (tentacles retraction and budding)

^b^*Daphnia magna*: LC50 based on mortality; EC50 based on immobilization (absence of swimming activity)

^c^95% confidence interval. Data are expressed as nominal concentrations

Analysis of gene expression revealed important changes at low threshold concentrations ( < 0.3 µg/L). The selected genes targeted critical physiological functions, such as protein recycling and autophagy (*MANF* and *MAPCI3*), oxidative stress (*SOD, CAT*), DNA repair of oxidatively damaged guanine (*OGG*) and governing cell differentiation and regeneration (*SRF1*; Hoffmann and Kroiher, 2001). The toxicity thresholds for gene targets are reported in Table 3. For Pd, 5/7 genes were affected at low threshold concentrations, some at concentrations lower than 0.3 µg/L (*MAPCI3, SERF1* and *OGG*). Genes expressions were significantly reduced for *MANF, MAPCl3*, *SOD, CAT* and *SRF1* at concentrations below the appearance of sublethal morphological changes (0.3 and 5.5 µg/L, Fig. 1). For Pt, 4/7 gene targets were influenced at higher concentrations, with threshold concentrations ranging from 5.5 to 17.6 µg/L. Furthermore, the gene expression changes followed a non-monotonic trend, with the significant induction of several genes (*MANF, MAPC13, SOD* and *OGG*) at the lowest concentration (0.3 µg/L), followed by a decrease at higher concentrations. For Rh, only 2/7 genes were significantly modulated by exposure (*MAPCI3* and *OGG*), although oxidative DNA damage repair (*OGG*) was increased at threshold concentrations <0.3 µg/L. Similarly, as for Pt, non-monotonic responses were also found for 5 genes (DDC, MANF, MAPCI3, SRF1 and OGG), whereas the other 2 were suppressed at the lowest concentration followed by an increase at higher concentrations. *CAT* gene expression was transiently increased at 10 µg/L. For Ru, 4/7 of gene targets were influenced at the threshold concentration between <0.3 to 17.6 µg/L. The most often affected gene transcripts were *MANF, MAPCI3* and *OGG*, suggesting alterations in protein integrity and DNA repair activity of oxidized DNA adducts. *DDC* and *SFRI* were significantly decreased at 31 µg/L, whereas *MANF* gene expression was first reduced at the lower concentrations, only to increase at 31 µg/L. For *MAPCI3*, a transient decrease was observed at 10 µg/L, and *SOD* gene expression was significantly reduced as well.

**Table 3:** Threshold concentration of gene expression changes in *Hydra Vulgaris*

Elements	Pd (µg/L)	Pt (µg/L)	Rh (µg/L)	Ru (µg/L)	# Elements
DDC	nd^a^	nd	nd	17.6	1/4
MANF	5.5	17.6	nd	<0.3	3/4
MAPCI3	<0.3	5.5	5.5	17.6	4/4
SOD	5.5	nd	nd	5.5	2/4
CAT	nd	17.6	nd	nd	2/4
SRF1	<0.3	nd	nd	17.6	2/4
OGG	<0.3	5.5	<0.3	nd	3/4
**Hydra EC20**	23	21	nd ( > 100 µg/L)	19	
**# genes affected**	5/7	4/7	2/7	4/7	

^a^Calculated based on the threshold concentration (nominal): (Lowest significant effect concentration X No effect concentration)^1/2^

**Fig. 2:**
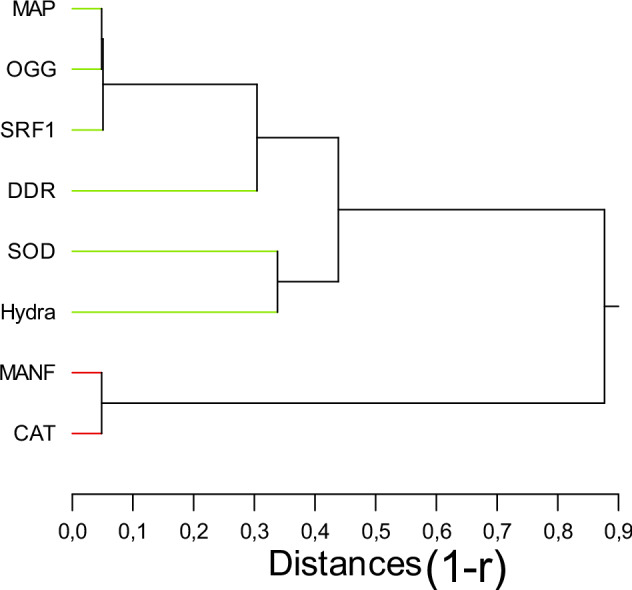
Hierarchical tree analysis of gene expression and morphological changes in hydra. Hierarchical tree analysis was performed using the (1-Pearson correlation r) measure for distance. The EC20 was taken for the hydra toxicity data

In the attempt to highlight which genes were closely related to sublethal morphological effects, a hierarchical tree analysis was performed (Fig. 2). The analysis revealed that morphological changes were associated with decreased gene expression in *SOD* with a significant correlation (r = 0.67, p < 0.05). Protein damage recycling (autophagy), regeneration and neural activity (*DDC*) was closely related to oxidative markers, such *CAT* and *OGG*.

## Discussion

The toxicity of the PGE was examined using a multispecies test battery in bacteria, algae, daphnids and hydranths. More detailed sublethal effects were provided in hydra at the gene expression level to highlight the molecular changes that precede morphological changes with the exception of Ir since it was reported at sub ng/L levels in wastewaters (Jackson et al., 2010). The toxicity of the most abundant PGE with the above organisms were solely performed for comparative purposes in respect to lethal and sublethal effects observed in hydra. As such, the discussion is focused on the toxicity responses found in hydra. Although these elements are generally less soluble than rare earth elements, their stability in aquarium water was studied to better understand their bioavailability and toxicity in both daphnids and mussels (Singer et al., 2005; Zimmerman et al., 2017). For instance, the measured value of Pd, Pt and Rh at 500 µg/L were found between 80–110% in aquarium water. The concentrations decreased to 10% after 3 days (before medium change) in the presence of zebra mussels, and significant bioaccumulation was found, with bioconcentration factors ranging from 70 to 1390 (Pd). The exposure concentration used was 5-fold higher than the concentration used in the present study. Pd, Pt and Ru were seemingly equally toxic based on the EC20 observed in hydra, whereas Rh was not toxic. Rh was also the least bioavailable element in zebra mussels compared to Pd, and Pt (Singer et al., 2005). This suggests that the lack of toxic effects in hydra could be due to a lower bioavailability, as observed in mussels. However, Rh was able to reduce gene expression in protein autophagy and DNA repair of oxidized guanine at concentrations of 0.3 and 10 µg/L in the present study, suggesting gene expression effects that were not morphologically observable. Overall, the thresholds for gene expression were smaller for Pd compared to Pt, Rh and Ru. It is noteworthy that the marine bacteria bioassay did not respond to the PGE in the present study. Possible reasons for this included marine bacteria that were either less susceptible to the toxicity of these elements, or that these elements were not bioavailable because of the high salt content in the marine exposure media (in the order of 30 g of salts/L). Indeed, Romero-Freire et al., (2021) have shown that specific speciation equilibrium, which is influenced by media composition, strongly impacts the low-kinetic of Pt, hence influenced its toxicity to the microalgae *Dunaliella salina*. Another study has shown the importance of Pt speciation in relation to its toxicity to *Pseudomonas aeruginosa* (Aruguete et al., 2023), highlighting the importance of the exposure media in bioavailability of PGE to bacteria.

Pt, Pd and Rh were reported to increase heat shock proteins response (hsp70) in the freshwater mussel *Dreissena polymorpha*, suggesting protein toxicity and reticulum endoplasmic stress at the salvage and removal steps of denatured proteins (Singer et al., 2005). Interestingly, the threshold level for hsp70 induction were lower for Rh although it was less bioavailable, and was followed by Pd and Pt. The highest hsp70 levels were obtained in mussels exposed to Pd (25-fold increase) followed by Pt and Rh (19-fold increase) (Singer et al., 2005). The toxicity of Ru was much less investigated in the science databases. Organic complexes of Ru (ruthenium red) are well known calcium uptake antagonists, by inhibiting Ca^2+^-ATPases (Liu et al., 2018). For instance, tolerance of *Limnodynastes terraereginae* embryos to low pH was improved by the addition of calcium and ruthenium red in water (Hird et al., 2022).

Oxidative stress was observed in hydra by changes in *SOD*, *CAT* and *OGG* gene expression. The elements Pd, Pt and Rh were found to induce oxidative stress in other species as well, triggering, for instance, morphological changes and reduced growth in *Lemna minor* at a concentration range of 0.5–5 mg/L (Bednarova et al., 2014). They obtained a 168 h EC50 of 2.3 mg/L for Pt, 5 mg/L for Pd and 2.5 mg/L for Rh. The levels of oxygen radicals (as determined by the ferric reducing antioxidant power methodology and ABTS reducing potential) were readily increased with Pt, suggesting the release of radicals in exposed organisms. This is consistent with the initial increases in *SOD* gene expression by Pt in hydra.

The decrease in *OGG* gene expression suggests that these elements were able to reduce the excision repair of 8-oxoguanine DNA, which could increase the susceptibility of organisms towards the accumulation of oxidized DNA and subsequent clastogenic effects (micronuclei/chromosomal aberrations). *OGG* gene expression was reduced at relatively low concentrations for Pd, Pt and Rh but not by Ru. *OGG* gene expressions were significantly correlated with ubiquitin and autophagy pathways (*MANF* and *MAPCI3*) and *SOD* suggesting protein degradation and oxidative stress in hydra. *OGG* gene expression in hydra exposed to Rh were related to protein damage, ubiquitin tagging and removal (*MANF, MAPC13*). This agrees with *Marisa cornuarietis* snails exposed to PtCl_2_, leading to elevated DNA damage using single cell electrophoresis at concentrations of 1 µg/L (Osterauer et al., 2001; 2009) In human cell cultures, Pd induced DNA strand breaks at 1 mg/L (Viau et al., 2021); Pd (II) also led to the highest ratio in double strand damage/micronuclei formation in human cells. In human lymphocytes, Pt and Rh strongly increased micronuclei formation compared to Pd, suggesting different genotoxicity potential and mechanisms (Migliore et al., 2002). Single cell electrophoresis following pretreatment with either endonuclease III or formamidopyrimidine glycosylase revealed that Rh increased oxidative damage to DNA, indicating that other elements, such as Pd and Pt, also induced DNA damage via clastogenic and aneuploidogenic mechanisms (*i.e*. not solely via oxidation). Using a bacterial DNA repair assay, Pt and Rh compounds were able to induce oxidized DNA repair (*OGG*), although it was increased only by 1.1–1.43 fold for Pt, and Rh was therefore considered weakly genotoxic (Gebel et al., 1997; Lantzsch and Gebel, 1997). While Ru did not produce any significant increase in *OGG*, it was found to increase the effects of electromagnetic radiofrequencies on DNA damage (Sun et al. 2023). Ru is known to inhibit a mitochondrial calcium uniporter, leading to the accumulation of free calcium, oxidative stress and apoptosis initiation. Hence, the genotoxicity of Ru cannot be ignored. In another study, gel electrophoresis analysis revealed that Ru (II) and Ir (III) chloride salts did not clearly damage plasmid DNA (Komarnicka et al., 2022). However, metal complexes (Ru(η6-p-cymene)) were able to stimulate reactive oxygen species, which can oxidize DNA or initiate apoptosis. An Ru complex of tryptophan and methionine led to cell division arrest and apoptosis (caspase 2 and Annexin V) in breast cancer cells (Mello-Andrade et al., 2022). Finally, the ionic form of Pt was shown to bind more strongly to plasma proteins than an organic complex of Pt, suggesting that proteotoxicity could be a common adverse outcome pathway for the PGE (van der Vijgh and Klein, 1986). This was corroborated by the observation that all 4 elements produced changes in the ubiquitin proteasome pathway (*MAPCI3* and *MAPCl3* gene expression) in hydra. For example, the binding constants of the Ru complex [Ru(II)(dcbpy)_2_Cl_2_] to albumin, phospholipase 2 and glutathione were 1.8 × 10^3^, 7.7 × 10^5^ and 2.5 × 10^2^ M^−1^, respectively (Nešić et al. 2016). This indicates that Ru has relatively low thiol binding affinity in respect to other proteins, such as phospholipase and albumin.

In conclusion, the lethal and sublethal toxicities of 4 PGE (Pd, Pt, Rh and Ru) were examined in bacteria, algae, daphnids and more extensively in *Hydra vulgaris*. Although no lethal effects were observed after 96 h exposure to 100 µg/L in hydra, morphological sublethal effects were found for Pd, Pt and Ru at around 20 µg/L corresponding to concentrations 300 x higher than those reported in municipal effluents (Jackson et al., 2010). Sublethal effects were also examined at the molecular level (gene expression) and revealed changes for all tested elements at threshold concentrations reaching sub µg/L, corresponding to concentrations close to those found in municipal effluents. This suggests that sublethal effects are likely in organisms living near urban areas. Changes in gene expression in protein recycling/autophagy, oxidative stress (*SOD*, *CAT*) and DNA repair of oxidized DNA (*OGG*) were observed for most of these elements. In conclusion, the occurrence of PGE at the sub µg/L could lead to toxic effects in hydra. The induced changes in gene expression at concentrations below the appearance of any morphological changes suggest that chronic exposure to low concentrations could induce effects and toxicity on hydra populations at the long-term scale.

## Supplementary information

Supplementary material

## Data Availability

Data is available upon request to the corresponding author.
