# Supplementary material for: Multiple species toxicity of selected platinum group of elements: focusing on Hydra vulgaris gene expression responses
Source: Ecotoxicology. 2025 Jul 29;34(9):1691–700. doi: 10.1007/s10646-025-02942-4 (PMC12553595; doi:10.1007/s10646-025-02942-4)
Supplement: Supplementary file 1 — Supplementary material [file 10646_2025_2942_MOESM1_ESM.docx]

Supplementary material

| 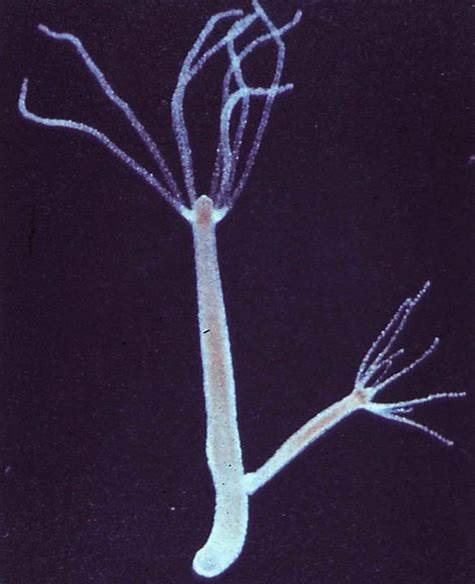 | 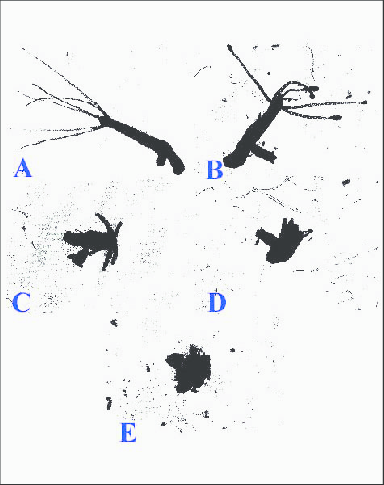 |
| --- | --- |

Figure 1S. Characteristic morphological changes in Hydra under toxic stress.

Normal A, tentacle contraction and budding B, severe contraction C; tulip stage D and desintegrated E.

Table 1S. Physico-chemical characteristics^1^

| Éléments | MW  (g/mol) | Ionic radius  (pm) | Electronegativity  (Pauli scale) | Reduction potential (Eo/V) |
| --- | --- | --- | --- | --- |
| Ir(III) | 192.22 | 75 | 2.2 | 1.156 |
| Pd (II) | 106.4 | 64 | 2.2 | 0.915 |
| Pt (IV) | 198.1 | 70 | 2.28 | 1.045 |
| Rh (III) | 102.9 | 84 | 2.28 | 0.76 |
| Ru (III) | 101.7 | 65 | 2.2 | 0.249 |

1. From Emsley, 1989.

| Nominal added  concentration  (µg/L) | Pd  (µg/L)  1-96h | Pt  (µg/L)  1-96h | Rh  (µg/L)  1-96h | Ru  (µg/L)  1-96h |
| --- | --- | --- | --- | --- |
| Control | nd-nd^1^ | nd-nd | nd-nd | nd-nd |
| 0.1 | 0.08-0.09 | 0.1-0.1 | 0.1-0.1 | 0.1-0.1 |
| 0.3 | 0.21-0.19 | 0.25-0.28 | 0.34-0.33 | 0.3-0.3 |
| 1 | 0.6-0.55 | 0.89-0.87 | 1-1 | 0.95-0.95 |
| 3 | 2-2 | 2.8-2.8 | 3.2-3.1 | 3.1-3.1 |
| 10 | 7.9-7.2 | 8-8.5 | 10-10 | 9.8-9.8 |
| 32 | 27-28 | 27-28 | 31-31 | 31-32 |
| 100 | 88-90 | 87-88 | 99-96 | 97-99 |

Table 2S Stability of selected elements in the hydra media.

1. non-detected at a detection limit of 0.005 µg/L for these elements.

References

Emsley, J., 1989. The Elements. Oxford University Press, pp. 257 (ISBN 0-19-855238-6).
